# Heated communities: large inter- and intraspecific variation in heat tolerance across trophic levels of a soil arthropod community

**DOI:** 10.1007/s00442-017-4032-z

**Published:** 2017-12-09

**Authors:** Oscar Franken, Milou Huizinga, Jacintha Ellers, Matty P. Berg

**Affiliations:** 1 Section of Animal Ecology, Department of Ecological Science, Vrije Universiteit, Amsterdam, De Boelelaan 1085, 1081 HV Amsterdam, The Netherlands; 2 Groningen Institute for Evolutionary Life Sciences, Community and Conservation Ecology Group, University of Groningen, PO Box 11103, 9700 CC Groningen, The Netherlands

**Keywords:** Critical thermal maximum (CT_max_), Extreme event, Heat wave, Functional trait, Thermal stress

## Abstract

Temperature extremes are predicted to increase in frequency, intensity and duration under global warming and are believed to significantly affect community composition and functioning. However, the effect of extreme climatic events on communities remains difficult to predict, especially because species can show dissimilar responses to abiotic changes, which may affect the outcome of species interactions. To anticipate community responses we need knowledge on within and among species variation in stress tolerance. We exposed a soil arthropod community to experimental heat waves in the field and measured heat tolerance of species of different trophic levels from heated and control plots. We measured the critical thermal maximum (CT_max_) of individuals to estimate inter- and intraspecific variation in heat tolerance in this community, and how this was affected by experimental heat waves. We found interspecific variation in heat tolerance, with the most abundant prey species, the springtail *Isotoma riparia*, being more sensitive to high temperatures than its predators (various spider species). Moreover, intraspecific variation in CT_max_ was substantial, suggesting that individuals within a single species were unequally affected by heat extremes. However, heat tolerance of species did not increase after being exposed to an experimental heat wave. We conclude that interspecific variation in tolerance traits potentially causes trophic mismatches during extreme events, but that intraspecific variation could lessen these effects by enabling partial survival of populations. Therefore, ecophysiological traits can provide a better understanding of abiotic effects on communities, not only within taxonomic or functional groups, but also when comparing different trophic levels.

**Electronic supplementary material:**

The online version of this article (10.1007/s00442-017-4032-z) contains supplementary material, which is available to authorized users.

## Introduction

Most terrestrial ecosystems irregularly experience sudden and severe changes in environmental conditions, for example, due to extreme weather events, such as heat waves, drought spells, and floods. Such extreme events have recently received increasing experimental attention (e.g., De Boeck et al. 2011; Krab et al. 2013; Kayler et al. 2015), but their impact on the community relative to more gradual changes in abiotic conditions remains hard to assess. Whereas, extreme events occur infrequently and usually last for a short period of time, they impose more extreme conditions on organisms and the sudden onset of these events leaves little time for adaptation. Moreover, current climate change scenarios predict an increase in frequency, intensity and duration of extreme weather events (Easterling et al. 2000a, b; Beniston et al. 2007; Rahmstorf and Coumou 2011), which may amplify their ecological impact (Gutschick and BassiriRad 2003; Jentsch et al. 2007).

A major challenge in community ecology is to predict the effects of such extreme weather events on community structure and composition (Gilman et al. 2010; Sutherland et al. 2013). The main difficulty in anticipating a community response is that different species within the community can show dissimilar responses to abiotic stress, which may be due to differences in physiological tolerances or behavioural strategies and microhabitat use to avoid stress. In addition, the variation among species in their responses to environmental change may result in altered trophic, competitive or facilitative interactions and interaction strengths (Tylianakis et al. 2008; Walther 2010; Berg et al. 2010). To anticipate community responses we need knowledge on interspecific and intraspecific variation in responses of species to abiotic stress, which can be investigated using functional traits that relate to species tolerance.

Trait-based approaches have been advocated as a more mechanistic approach to foresee how communities are affected by environmental change (McGill et al. 2006; Langlands et al. 2011; Dias et al. 2013). Multiple studies have related variation in tolerance traits and their environmental drivers to spatial distribution of species (Dias et al. 2013; van Dooremalen et al. 2013), (dis)assembly processes (Lindo et al. 2012; Bartlett et al. 2016), and community composition (Bokhorst et al. 2012). However, trait studies have mainly been carried out within single functional groups or trophic levels (e.g., Dias et al. 2013; van Dooremalen et al. 2013), rather than across trophic levels within a single community (but see Sentis et al. 2013; Puentes et al. 2015). Moreover, studies have predominantly used mean trait values of species to characterize interspecific variation in tolerance, ignoring the fact that trait distributions between species may overlap due to intraspecific variation of trait values (Albert et al. 2011; Violle et al. 2012). Intraspecific variation in stress tolerance determines the relative proportion of a population that will be affected by the extreme conditions and the proportion that can still interact with other species. Thus, including within-species variation in trait-based studies predicting community responses is imperative, as previously shown for Collembola (Janion et al. 2009).

Intraspecific variation of trait values can be caused by several underlying mechanisms, and these may differentially affect persistence under extreme conditions. For instance, many species show ontogenetic variation in traits related to abiotic stress, which affects survival probabilities of different life stages (Bowler and Terblanche 2008; Hoffmann 2010; Kingsolver et al. 2011; Nakazawa 2014; Zizzari and Ellers 2014). Especially when extreme conditions affect only a particular age or size class, such as small-sized juveniles or reproductively active adults, severe consequences for the population can be expected. This may alter size distributions and population dynamics with cascading effect on higher trophic levels (e.g., through reduced prey density) or lower trophic levels (e.g., through reduced predation pressure). Another important source of intraspecific variation in trait values is phenotypic plasticity (DeWitt and Scheiner 2004), by which previous exposure to stressful conditions may lead to a rapid increase in tolerance to subsequent exposures, which may even act across generations (e.g., Bateson et al. 2014; Zizzari et al. 2016). Moreover, if species differ in the degree to which they adjust their physiology to extreme conditions, this may differentially affect species’ survival when exposed to extreme events, thereby affecting the interaction strength between the species. Understanding the causes and consequences of intraspecific variation can provide valuable insight in the effects of extreme events at the population level, and subsequently on the interactions with other species in the community.

In this paper we investigate the extent of inter- and intraspecific variation in heat tolerance of several species from a soil arthropod community that was exposed to experimental heat waves. These heat waves were induced by placing small plastic greenhouses with heat emitters in the field. We focus on a thermal trait that is related to temperature extremes: the critical thermal maximum (CT_max_), which reflects the heat tolerance of an individual when exposed to a gradually increasing temperature (Lutterschmidt and Hutchison 1997; Terblanche et al. 2007; Mitchell and Hoffmann 2010). As CT_max_ is measured at the individual level, this tolerance proxy allowed us to measure intraspecific variation in heat tolerance and link this to body size effects. Using species from a single arthropod community, we maximize the likelihood that they have previously experienced the same climatic conditions, although the actual habitat mean temperature and variance that individuals have experienced is likely to be modified by behavioural differences and microhabitat use.

We expected to find interspecific differences in heat tolerance due to differences in physiology among species, especially because thermal traits are often considered to be phylogenetically conserved (e.g., Kellermann et al. 2012; Araújo et al. 2013). We further expected large intraspecific variation within the measured species, mostly due to differences in body size and associated ontogenetic variation. Specifically, we hypothesize that larger, adult individuals are more sensitive to exposure to high temperatures than smaller, juvenile individuals (Bowler and Terblanche 2008; Zizzari and Ellers 2014). Finally, we expected an increase in heat tolerance of species after being exposed to an artificial heat wave in the field, either because of phenotypic plasticity in heat tolerance or because of disproportional survival of more tolerant individuals. We will discuss the ecological consequences of the inter- and intraspecific variation in heat tolerances in terms of species interactions within our soil community.

## Materials and methods

### Field site

Our experiment was conducted on the southwest side of the barrier island of Schiermonnikoog (53.46896°N, 6.13914°E), The Netherlands. The ecosystem consisted of a vegetated beach which was characterized by a rather low diversity of plant species and a relatively low species richness of the arthropod community compared to other terrestrial ecosystems (van Wingerden and den Hollander 1981; Ellers et al. 2011). This allowed us to collect individuals from all the dominant taxa living on the soil surface and include them in our study. The study site was located ~ 400 m north of the mean high tide line, in an area which annually received about five to ten sea water inundation events. During our experiment, there were no inundation events. The vegetation was dominated by *Glaux maritima* L. with tufts of *Carex extensa* Gooden, *C. distans* L. and *Limonium vulgare* Mill. Approximately the first cm of the soil consisted of sand mixed with fine organic matter with almost no build-up of a leaf litter layer. In patches where vegetation cover was sparse, a microbial mat covered the soil surface (Bolhuis et al. 2013). In 2014, the average annual temperature was 11.5 °C, with a measured summer maximum of 34.4 °C. There were 8 days in 2014 on which the temperature exceeded 25.0 °C, and an additional 67 days with temperatures exceeding 20.0 °C. Annual precipitation was 621.8 mm, making 2014 a warmer and dryer year compared to long term averages (all climate data from weather station ‘Lauwersoog’, The Netherlands. Source: http://cdn.knmi.nl/knmi/map/page/klimatologie/gegevens/mow/jow_2014.pdf).

### Artificial heat waves in the field

We established four spatially separated plots which were aligned parallel to the dune ridge and the sea. Plots were chosen to have similar elevation above mean sea level and, therefore, comparable inundation regimes and vegetation types (Fig. S1). The distance between plots ranged from ~ 15 to ~ 80 m to reduce spatial variability over larger scales. Within these four plots, four subplots of 70 × 70 cm each were created, resulting in a total of 16 subplots. Our experimental capacity was limited to four heating units; therefore, the experiments were carried out in two periods (period 1 from the 3rd to the 10th of June 2014 and period 2 from the 20th of July to the 7th of August 2014). For each plot the four subplots were randomly assigned to a combination of period and treatment (control period 1, treatment period 1, control period 2, treatment period 2).

To heat the soil in the treated subplots, we covered them with miniature greenhouses (hereafter tents) made out of transparent polythene sheets (Botanico PNI1101) (Fig. S1). Sheets were placed over two crossed plastic pipes (ø 1 cm) of ~ 157 cm length that formed two halve-circle arcs with a maximum height of 50 cm above the soil. Two ceramic heat emitters (60 Watts each, RepTech) were attached on the pipes, each 12.5 cm from the center were the pipes crossed. Heat emitters were placed 20 cm above the soil surface and were powered to their full capacity by a generator (Honda EU10i). Tents were set-up for 4 h between ~ 11:00 and 15:00 to specifically mimic an increase in maximum temperature during the hottest period of each day. Using this method, the treatment subplots were warmed relative to the control subplots, even though absolute temperatures reached were dependent on the ambient conditions. During heating, the base of the tents were pressed to the ground by placing a heavy iron chain on top of a 3–5 cm wide band of sheet around the subplots, to limit movement of animals out of the plots. The tents remained on the subplots for an additional hour with the heat emitters turned off to allow the air to cool down gradually. Control subplots were not covered by tents and exposed to ambient field conditions. Compared to the control subplots, the heating resulted in an average increase of the maximum temperature of 7.4 °C for period 1 and 4.0 °C for period 2 (Table 2 and Fig. S5).

Since the tents were removed daily, temperatures dropped to ambient night temperatures, whereas during natural heat waves it is not uncommon that also night temperatures are relatively high. However, nighttime exposure was not included in this experiment as it could have introduced unwanted artefacts such as a high relative humidity. By removing the tents, animals possibly moved in or out of the subplots during the night, potentially diluting the effect of heating. However, soil organisms generally have a small home range, as was the case for our species. More specifically, the genus of the most abundant prey species in our study, *Isotoma* (Collembola) moves only ~ 5 cm week^−1^ (Ojala and Huhta 2001), and the most abundant predator species, i.e., web building dwarf spiders (Linyphiidae), can be considered largely sedentary. Lycosid spiders were the most mobile species, but mark-recapture studies shows only ~ 20% probability to move to an adjacent plot (1 m) in 4 days (Ahrens and Kraus 2006).

Temperature was recorded to the nearest 0.01 °C every minute using temperature loggers (Tinytag Plus2) of which the sensors were placed horizontally on the soil surface in both control and heated subplots. Temperature profiles were retrieved with the program EasyView (Version 5.7.0.1. © INTAB Interface-Teknik AB 2002) and data were exported to Microsoft Excel (Microsoft Office 365 version 1611) for further analysis. Every day, the average and maximum temperature was calculated for both the control and heated subplots of a selected plot pair. Per subplot, the average of the temperature data of the exposure days was used for statistical analysis. A paired *t* test was performed in Microsoft Excel to test for effectiveness of the heat treatment.

In the first experimental period we applied a single heat wave of 5 consecutive days, which resembles a meteorological heat wave according to The Royal Netherlands Meteorological Institute (https://www.knmi.nl/kennis-en-datacentrum/uitleg/hittegolf, in Dutch). We used a staggered exposure period, starting with the exposure of one pair of subplots (control and treatment), and adding one random pair to the exposure every day. This design allowed us to harvest a single pair of subplots per day (which was necessary due to logistic constraints, see below), while each pair still had 5 days of exposure. Preliminary analysis showed no effect of this single heat wave on the thermal sensitivity of the soil organisms. Therefore, in the second experimental period we applied two consecutive artificial heat waves of 5 days each to further increase thermal stress. We are aware that this experimental design does not allow us to disentangle effects of the experimental period and number of heat waves, therefore the combined effect of experimental period and number of heat waves was included as a random effect in the statistical analyses to account for any introduced variation by these combined effects (see Supplemental information 2). In total, this design resulted in eight heated and eight control subplots for the full experimental period.

### Critical thermal maximum (CT_max_)

To obtain samples for the measurement of CT_max_, animals were collected from the control and heated subplots directly after the removal of the tents. Vegetation and top soil in the subplots was carefully, manually searched for 1 h. The arthropods (i.e., springtails, spiders and insects) were collected alive using a pooter and stored in plastic containers (ø 13.1 cm, height 5.8 cm) with a ~ 0.5 cm thick bottom of water-saturated plaster of Paris and some vegetation material. Predators were collected in separate containers from the prey to prevent predation. The containers were transported to the laboratory within an hour.

For the measurement of CT_max_ animals were placed individually in a glass jar (ø 2.7 × 7.5 cm) with a ~ 2.1 cm layer of water-saturated pink plaster of Paris (Regal Die Violet class 4, Gips Zwolman) to prevent dehydration. Twenty-five jars were placed in a Styrofoam plate (37.5 × 29.9 × 2.0 cm) in a 5 × 5 grid with ~ 2.3 cm intervals. This plate was placed in a 19 L water bath (Julabo Bath tank 19) with a heating immersion circulator (Julabo MB) (Fig. S3). The water level in the bath was set to come up to the first 0.5 cm of the Styrofoam plate. The bottom of the vials protruded ~ 2.1 cm from the underside of the Styrofoam plate to facilitate heat conduction from the water to the layer of plaster in the jar. Animals from different treatments or size classes were always mixed within a single run, to prevent bias between runs, and were randomly assigned to the 24 jars. Three runs per pair of control and heated subplots were needed to test the collected animals for CT_max_, resulting in measurements of a maximum of 72 individual animals per day. The water was heated following a ramping assay from an initial temperature of 20 °C, with a steady increase of 0.35 °C min^−1^ (Julabo EasyTemp software version 3.4.1). The initial temperature of 20 °C was close to the soil temperatures at time of sampling, and chosen to standardize measurements over the different days. During each run a logger (iButton, Maxim Integrated DS1923) was placed in the center jar of the plate to monitor changes in moisture and temperature. The programmed increase of 0.35 °C min^−1^ of the water resulted in a 0.33 °C min^−1^ steady increase within the test vials. This rate was relatively fast compared to other studies, which are typically in the range of 0.06–0.25 °C min^−1^ (Terblanche et al. 2007; Moretti et al. 2017), but this rate was chosen to complete three water bath runs per day, which was essential to process all animals per subplot pair within a day. CT_max_ values are known to depend on methodological context (e.g., Terblanche et al. 2007; Chown et al. 2009; Mitchell and Hoffmann 2010), with higher ramping rates giving rise to higher estimates of CT_max_ (because of shorter total exposure time until death). Although the absolute value of CT_max_ measured for each individual in our study may partly result from the chosen ramping rate, differences between individuals or species were meaningful because the same methodology was applied throughout the experiment. A correction was applied to correct the recorded water temperature to the air temperature in the vials (Fig. S4).

Animals in the vials were observed continuously during the gradual increase of the air temperature. CT_max_ was recorded as the temperature at which there was no movement of appendages of the animal observable after gently touching them with a fine brush. We chose this protocol to standardize the method for use of a diversity of taxa within one measurement (for a discussion on possible endpoints in CT_max_ measurements see Lutterschmidt and Hutchison 1997).

After the CT_max_ measurements, the animals were fixed in 70% ethanol and stored for species identification and body size measurements. For body size measurements we used a mounted camera (Olympus SC30) on a Leica Wild M8 stereomicroscope (with 6× magnification) to make digital images of each individual. During measurement, the animals remained submerged in 70% ethanol. Body size was measured from these images using cellSens Entry 1.7 software (Olympus). Collembola were measured dorsolaterally, following and including the curvature that normally occurs when storing some Collembola in ethanol. Hemiptera, Coleoptera and Araneae do not show this altered body shape and were measured dorsally, to the nearest µm. For spiders it is also common to use the length or width of the cephalothorax, but we refrained from using these measurements to be able to directly compare the body sizes of all taxonomic groups.

### Analysis and statistics

Statistical analyses were performed using R version 3.3.1 (R Core Team 2016), which was run in the RStudio interface (RStudio Team 2015). Species with fewer than three individuals were not included in any of the analyses. We ran linear mixed effects models on the CT_max_ data using the package “lme4” (Bates et al. 2015) with the random factors: plot (spatial difference between the plots, linking the subplot pairs), experiment (experimental heating period 1 vs period 2) and run (temporal variation between water bath runs within a pair of subplots). To test whether the fit of the statistical models was improved when including interactions between the main factors, or to test if a main factor improved the model fit, we obtained Wald Chi-square statistics from the “Anova” function in the statistical package “Car” (Fox and Weisberg 2011).

First, we tested whether life stage and sex had significant effects on the observed CT_max_ values. The effect of life stage could only be assessed in the bug family Saldidae. Being hemimetabolous, both nymphs and adults live on the soil surface, and hence have comparable thermal environments throughout their life history. This does not hold for the holometabolous beetle family Heteroceridae, where a large part of the larval development takes place belowground. Also the spiders were not suitable for testing for life stage-related changes in CT_max_, since juvenile spiders could not be identified to the species level. Effect of sex on the observed CT_max_ was tested for the spiders *Erigone longipalpis* and *Oedothorax retusus*. The absence of a significant effect of the factors of life stage and sex would justify their omissions from further analysis.

We then tested whether the heating treatment and taxonomic group had a significant influence on the measured CT_max_ values, and whether this effect differed between taxa (Treatment × Taxon). Only the taxonomic groups for which we had data on more than ten individuals in the treatment and control subplots were included. Contrasts analysis was performed on significant fixed factors using the function “lsmeans” in the similarly named package (Lenth 2016), with a Tukey correction for multiple comparisons. To test for interspecific effects of body size on the observed CT_max_ values, we fitted a weighted least squares linear model to the average CT_max_ and average body size of all taxonomic groups, with the weight being the number of measured individuals per group. Besides this interspecific relationship between CT_max_ and body size, it is expected that variation within taxa might contribute to the intraspecific variation in CT_max_. We used the data of the taxonomic groups with more than ten measured individuals to fit a linear mixed effects model with taxonomic group, individual body size and their interaction (Taxon × Body size), to test their effect on CT_max_. When interactions between taxonomic group and body size were significant, we investigated this intraspecific pattern for each taxon. Assumptions of the applied tests were visually checked by plotting the residuals of the fitted models.

## Results

### Soil fauna community

In total, we measured the CT_max_ and body size of 552 individuals of the arthropod community living on the soil surface (Table 1). The most abundant taxonomic group was the Collembola, present with a single species *Isotoma riparia* (*N* = 255). Two Linyphid spider species were present in high numbers, i.e., *Erigone longipalpis* (*N* = 36) and *Oedothorax retusus* (*N* = 57). Juvenile Linyphiidae could not be determined to species level and were pooled together (*N* = 79). The Lycosidae were also abundant, but we caught mostly juveniles (*N* = 31) and only two adults. As the adults belonged to two different species of wolf spiders, they were excluded from further analysis. We also found several spider species with lower numbers of adults, i.e., *Walckenaeria kochi* (*N* = 3), *Argenna patula* (*N* = 6) and *Pachygnatha degeeri* (*N* = 4). Finally, two insect taxa were found: *Heterocerus* sp. (Coleoptera, *N* = 20) and Saldidae (Hemiptera, *N* = 31).

**Table 1:** An overview of the soil animals for which CT_max_ and body size were measured and included in the analysis

Species	Author	Taxon	Family	*N*	
				Control	Treatment
*Isotoma riparia*	(Nicolet 1842)	Collembola	Isotomidae	127	128
*Walckenaeria kochi*	(O.P.-Cambridge 1872)	Araneae	Linyphiidae	3	0
*Oedothorax retusus*	(Westring 1851)	Araneae	Linyphiidae	20	37
*Erigone longipalpis*	(Sundevall 1830)	Araneae	Linyphiidae	15	21
Linyphiidae (juvenile)		Araneae	Linyphiidae	41	38
*Pachygnatha degeeri*	Sundevall 1830	Araneae	Tetragnathidae	3	1
*Argenna patula*	(Simon 1874)	Araneae	Dictynidae	2	4
Lycosidae (juvenile)^a^		Araneae	Lycosidae	16	15
Saldidae		Hemiptera	Saldidae	14	17
*Heterocerus* sp.		Coleoptera	Heteroceridae	10	10
				251	271

*N* gives the number of individuals included in the analysis

^a^Combination of *Pardosa purbeckensis* F. O. Pickard-Cambridge, 1895 and *Pirata piraticus* (Clerck 1757)

Statistical analysis showed that the heating treatment had a significant effect on both the average and maximum temperatures in both treatment periods (Table 2, Fig. S5).

**Table 2:** Abiotic conditions in control versus heated subplots

Experiment	Average temp. (°C ± SD)	Max. temp. (°C ± SD)
Period 1: one heat wave		
Control	26.1 ± 1.6	31.1 ± 1.5
Treatment	32.7 ± 1.4**	38.5 ± 1.1**
Difference	6.6 ± 1.3	7.4 ± 1.9
Period 2: Two consecutive heat waves		
Heat wave 1		
Control	29.6 ± 0.7	32.2 ± 0.7
Treatment	33.1 ± 0.5***	36.4 ± 0.4***
Difference	3.5 ± 0.3	4.2 ± 0.4
Heat wave 2		
Control	30.1 ± 0.7	34.6 ± 0.7
Treatment	33.6 ± 0.9***	38.3 ± 0.8***
Difference	3.5 ± 0.2	3.7 ± 0.3

Temperature was recorded at the soil surface. Values were averaged over the 5 days of exposure per plot, which masks part of the variation of the actual daily differences in the field

* *P* < 0.05, ** *P* < 0.01, *** *P* < 0.001, paired *t* test (See Fig. S5 for details per plot pair)

### No effects of life stage and sex on CT_max_

The effect of life stage on CT_max_ was tested in the Saldidae. Our analysis of CT_max_ shows that including life stage did not significantly improve the fit of the model ($\chi_{(1)}^{2}$ = 0.035, *P* = 0.85, Fig. S6a).

Sexual dimorphism is a second factor that could potentially increase intraspecific variation in CT_max_ for some of the taxonomic groups. Of the two Linyphiidae species tested, *O. retusus* (*N* = 57) showed clear body size dimorphism, with larger females than males, whereas *E. longipalpis* (*N* = 36) did not show size dimorphism. However, the statistical models showed no significant effects of sex on CT_max_ for either of these species (*O. retusus*: ($\chi_{(1)}^{2}$ = 0.43, *P* = 0.51); *E. longipalpis*: ($\chi_{(1)}^{2}$ = 2.7314, *P* = 0.098), see also Fig S6b, c. Based on the lack of significance of life stage and sex in the tested species, these variables were not included in the remainder of the analyses.

### The effect of artificial heat waves on CT_max_

Seven taxonomic groups had a sufficiently large sample size (i.e., *N* > 10 in both treatment and control) to test our hypothesis that exposure to higher temperatures in the field will result in higher CT_max_ values. We found that there was no interaction between the two main effects Taxon and Treatment ($\chi_{(6)}^{2}$ = 4.0, *P* = 0.68). Moreover, only Taxon had a strong effect on the observed CT_max_ values ($\chi_{(6)}^{2}$ = 962.8, *P* < 0.001), and the heating treatments did not have an effect on the CT_max_ values ($\chi_{(1)}^{2}$ = 0.60, *P* = 0.44).

**Fig. 1:**
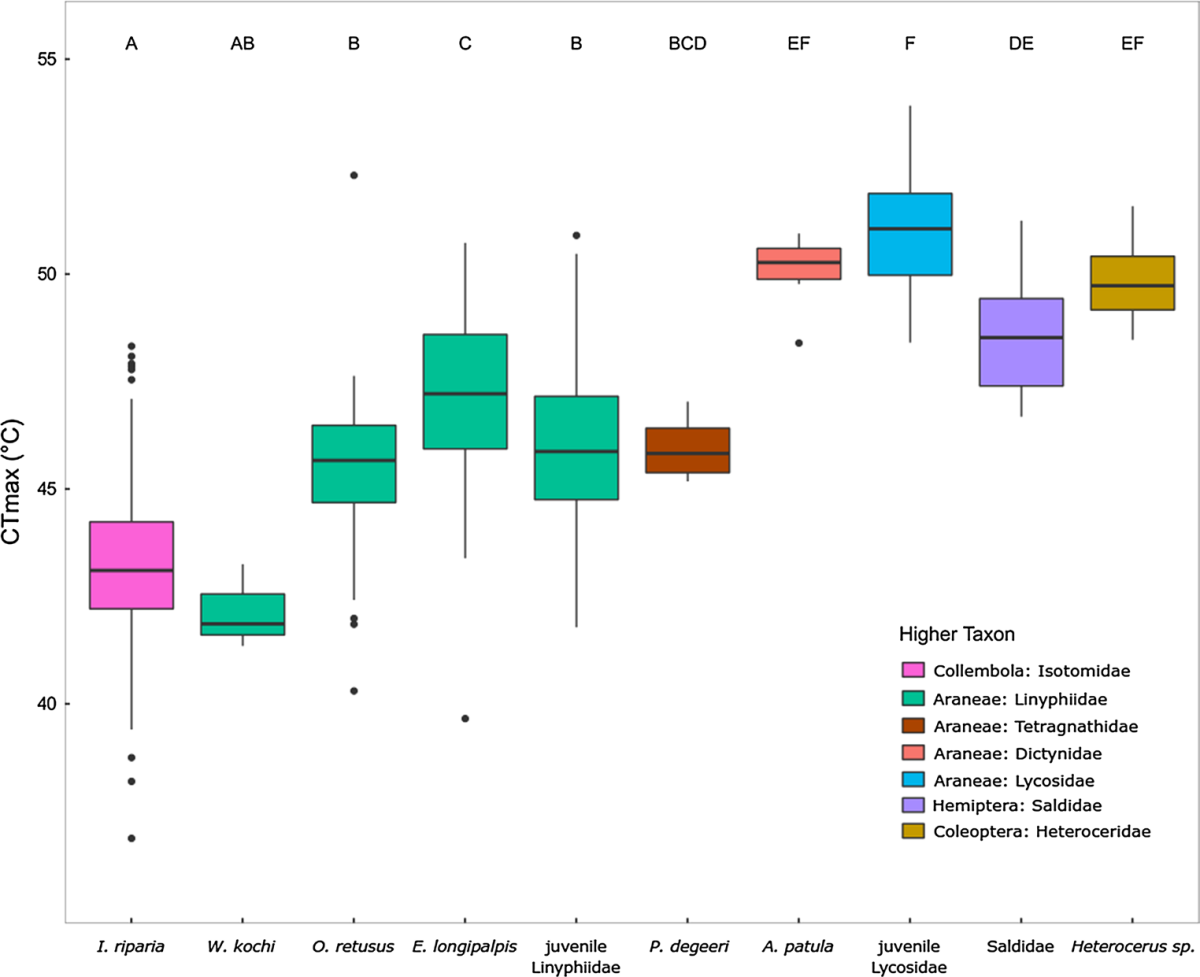
Differences in CT_max_ for the taxonomic groups included in our analysis. Boxplots indicate the median with quartiles, with whiskers indicating 1.5 times the interquartile range. In this analysis, data of the control and treatment subplots was combined, as there was no significant effect of the heating treatment on CT_max_ of the different taxa. For clarity, the different taxonomic groups are colour coded. Collembola and Heteroceridae are considered decomposers, while all spider species (Araneae) are predators. Saldidae are thought to either actively predate or feed on dead animals. For full species names see Table 1. Different letters indicate statistical difference at *P* < 0.05

As the heating treatments did not influence CT_max_ values, we ran the model again without treatment, this time including all available taxa. Taxonomic group remained highly significant in this analysis ($\chi_{(9)}^{2}$ = 1051.6, *P* < 0.001), and contrast analysis revealed large differences in CT_max_ between the taxonomic groups (Fig. 1). Especially the main prey species of our arthropod community, *I. riparia* (CT_max_ = 43.4 ± 1.8 °C) had a significantly lower heat tolerance than its most abundant predators, *O. retusus* (CT_max_ = 45.6 ± 1.8 °C; *t* ratio = 7.5; *P* < 0.001), *E. longipalpis* (CT_max_ = 47.1 ± 2.2 °C; *t* ratio = 12.7; *P* < 0.001), juvenile Linyphiidae (CT_max_ = 46.1 ± 1.9; *t* ratio = 10.5; *P* < 0.001) and the juvenile Lycosidae (CT_max_ = 51.1 ± 1.3 °C; *t* ratio = 22.8; *P* < 0.001; See Fig. 1 and Table 3 for details on the other taxa).

**Table 3:** Intraspecific variation in CT_max_ of the seven taxa with > 10 individuals in both control and treatment subplots

Species	Mean CT_max_	St. deviation	Range min–max CT_max_	Total variation in CT_max_
*I. riparia*	43.4	1.8	37.0–48.5	11.5
*O. retusus*	45.6	1.8	40.4–52.4	12.0
*E. longipalpis*	47.1	2.2	39.8–50.9	11.1
Linyphiidae (juv)	46.1	1.9	41.9–51.0	9.1
Lycosidae (juv)	51.1	1.3	48.5–54.0	5.5
Saldidae	48.6	1.3	46.8–51.4	4.6
*Heterocerus* sp.	50.0	0.9	48.6–51.7	3.1

Full species names are given in Table 1

### Inter- and intraspecific variation in CT_max_ and the effect of body size

To test if the observed interspecific differences in CT_max_ were related to variation in species’ body size, a linear model was fitted with average CT_max_ and average body size per taxonomic group, each weighted for the number of observations in that taxon. There was no significant relationship between average body size and average CT_max_ value of the taxa (*t*_(1,8)_ = 1.58; *P* = 0.153) (Fig. S7).

Besides interspecific variation, we also observed that CT_max_ varied considerably within each taxon. For example, CT_max_ of *I. riparia* ranged from 37.0 to 48.5 °C and the total variation in *O. retusus* was even higher: ranging from 40.4 to 52.4 °C (see Table 3 for all taxa). We tested intraspecific variation in body size as an explanatory variable for intraspecific variation of CT_max_ values. In addition to taxonomic group ($\chi_{(6)}^{2}$ = 942.4, *P* < 0.001), intraspecific variation in body size was also highly significant with an overall negative correlation between individual body size and CT_max_ ($\chi_{(1)}^{2}$ = 12.6, *P* < 0.001). However, there was a significant interaction between the factors taxon and body size ($\chi_{(9)}^{2}$ = 22.4, *P* = 0.001), indicating that the effect of body size on CT_max_ differed between taxonomic groups.

This interaction was further investigated by separately analysing the effect of individual body size on CT_max_ for seven taxa with sufficient sample sizes (the same taxa as used to test for artificial heating above, Table 4). We found that *I. riparia* and *Heterocerus* sp. had a significant negative relationship between CT_max_ and body size ($\chi_{(1)}^{2}$ = 17.3, *P* < 0.001 and $\chi_{(1)}^{2}$ = 9.5, *P* = 0.002 for *I. riparia* and *Heterocerus* sp., respectively). For every additional mm in body size, the CT_max_ was reduced by 0.82 and 0.92 °C for *I. riparia* and *Heterocerus* sp., respectively. Especially for *I. riparia* these values are substantial as body sizes ranged from ~ 1.2 to ~ 4.5 mm, resulting in a difference in thermal tolerance of almost 3 °C from the smallest to largest individuals. On contrary, the juvenile Linyphiidae showed a positive relationship between CT_max_ and body size ($\chi_{(1)}^{2}$ = 4.63, *P* = 0.032). This results in a 1.09 °C increase in CT_max_ for every additional mm of length.

**Table 4:** Results of the Wald Chi-square tests body size as the main effect in the fitted linear mixed effect models

CT_max_ (taxon) ~	Fixed and random factors	*χ* ^2^	*Df*	*P*	Slope
*I. riparia*	Body size + (1|Experiment) + (1|Run) + (1|Plot)	17.30	1	< 0.001	− 0.82
*O. retusus*	Body size + (1|Experiment) + (1|Run) + (1|Plot)	0.25	1	0.615	− 0.29
*E. longipalpis*	Body size + (1|Run) + (1|Plot)	0.43	1	0.513	− 0.92
Linyphiidae (juv)	Body size + (1|Experiment) + (1|Run) + (1|Plot)	4.63	1	0.032	1.09
Lycosidae (juv)	Body size + (1|Experiment) + (1|Run) + (1|Plot)	0.45	1	0.501	0.19
Saldidae	Body size + (1|Experiment) + (1|Run) + (1|Plot)	0.57	1	0.450	0.22
*Heterocerus* sp.	Body size + (1|Run) + (1|Plot)	9.49	1	0.002	− 0.92

The effect of body size on CT_max_ was tested for these seven taxa separately. Taxa for which CT_max_ was influenced by body size are displayed in bold. Full species names are given in Table 1

## Discussion

In this study we investigated inter- and intraspecific differences in heat tolerance among species of a soil arthropod community exposed to artificial heat waves. We found strong interspecific differences in heat tolerance, with the dominant Collembola species being more sensitive to high temperatures than its predators, mostly spider species (Fig. 1). Additionally, we observed large intraspecific variation in heat tolerance (Table 3), partly related to body size. Artificial heating of communities in the field, mimicking naturally occurring heat waves, had no detectable effect on heat tolerance of individual species. We discuss the ecological implications of inter- and intraspecific variation in heat tolerance on species interactions.

### Interspecific differences in heat tolerance

We observed significant differences in the level of heat tolerance between taxonomic groups. The prey species *I. riparia* had a significantly lower CT_max_ than most of its predator species, and also among predator species heat tolerance differed significantly. For example, within the spiders, the Lycosidae and Dictynidae had significantly higher CT_max_ values than the Linyphiidae and Tetragnathidae. Although interspecific differences in CT_max_ between taxa have been reported before (e.g., Addo-Bediako et al. 2000; Sunday et al. 2011; Araújo et al. 2013), this—to our knowledge—has never been reported for interacting species in a terrestrial community under field conditions. The information obtained from this approach therefore adds to studies focusing on specific interactions in the community (Sentis et al. 2013; Puentes et al. 2015) or studies comparing heat tolerances across spatial scales (e.g., Deutsch et al. 2008; Araújo et al. 2013).

The mechanisms underlying these interspecific differences in thermal tolerance are largely unknown. Previous research has already pointed to evolutionary constraints at the upper thermal limits of species (Addo-Bediako et al. 2000; Chown 2001; Mitchell et al. 2011; Kellermann et al. 2012; Hoffmann et al. 2013). However, a mechanistic explanation as to which trait is underlying these constraints is not evident and multiple factors can be simultaneously at play, such as membrane lipid composition (van Dooremalen et al. 2013), innate differences in heat shock protein expression (Belén Arias et al. 2011), oxygen limitation at higher temperatures (Klok et al. 2004), and interspecific differences in body size. Specifically the difference in body size is interesting considering that on the soil surface, which is heated by solar radiation, the smaller species in this community may experience higher temperatures compared to larger species because the smaller species’ bodies are closer to the heated soil surface (e.g., Kaspari et al. 2015). However, we found no significant relation between the average CT_max_ and body size of the taxa investigated here (Fig. S7). Measurements of both heat tolerance and these underlying traits in combination with phylogenetic analysis could potentially provide additional answers, but to do so more data are needed that are collected in a standardized and comparative way (Moretti et al. 2017).

The finding of substantial interspecific differences in heat tolerance within a single community, raises the question how these differences affect the dynamics of species interactions during and after heat waves. This is especially relevant as it is known that altered species interactions can pose a larger effect on communities than the direct abiotic effects (Ockendon et al. 2014). In line with the thermal mismatch hypothesis postulated for host-parasite interactions (Nowakowski et al. 2016; Cohen et al. 2017; Greenspan et al. 2017), our results indicate that thermal mismatches might also occur in predator–prey interactions in a community. Species with a high thermal tolerance are expected to be less affected by hot weather extremes than species with low thermal tolerance. Therefore, when such species co-occur, the species within communities will most likely not respond to climate change in similar ways (Petchey et al. 1999; Voigt et al. 2003).

The fact that higher trophic levels in our study seem to be more tolerant to high temperatures also highlights the difference in climate change effects of short term extreme events compared to more gradual and longer lasting time scales, as studies on the latter usually find that higher trophic levels are more sensitive to increases in temperature than lower trophic levels (e.g., Petchey et al. 1999; Voigt et al. 2003; Vasseur and McCann 2005; Gilman et al. 2010). This difference can be explained by our focus on the direct, physiological differences between the taxa, which is especially relevant when communities are exposed to extreme events. As such extremes can occur for one or several days (such as the 5 days used here), the stress is sudden and can directly cause differences in survival between species when their physiological thresholds are surpassed. On the contrary, the studies quoted above focus on longer time scales (even spanning multiple generation), and therefore on consequences for population dynamics. This emphasizes that the specific form of temperature changes that global warming can bring about (e.g., extreme event versus gradual change), can yield different predictions on the outcome at the community level.

### Intraspecific variation of thermal tolerance

In addition to interspecific differences in heat tolerance, within-species variation can also affect the outcome of species interactions. When intraspecific variation is large, some proportion of the individuals could survive and continue to interact in the community, depending on the severity of the heat wave. We found considerable levels of intraspecific variation in CT_max_ values of the prey species *I. riparia* (37.0–48.5 °C) and in some of the other taxa (see Fig. 1 and Table 3), with some of the individuals of interacting species showing overlap in tolerance trait values. Part of this intraspecific variation of CT_max_ may have resulted from variation in body size within taxa. We found a negative relationship of body size with CT_max_ in adult Heteroceridae and *I. riparia* (~ 1 °C lower for every 0.9 mm extra length). The degree of intraspecific variation of heat tolerance observed in our study is comparable to what is reported in existing literature, (e.g., Terblanche et al. 2005; Jumbam et al. 2008; Chown et al. 2009; Mitchell and Hoffmann 2010, when standard deviations are recalculated from reported standard errors). As we measured individuals straight from the field, without an acclimation period in the laboratory, we were able to obtain a nearly “complete estimation of within-species trait distribution” (sensu Violle et al. 2012) from the measured individuals in our community. We argue that to make statements on the effects of temperature on species performance and species interactions in natural ecosystems, heat tolerance should be measured directly from the field and encompass all relevant life stages of the species to prevent underestimation of trait variation. This is especially true when the intraspecific variation is important in understanding effects of climatic extremes on species interactions.

### No evidence for heat wave induced phenotypic plasticity of CT_max_

Phenotypic plasticity is a common way to mediate sudden adverse effects of changing environmental pressures (DeWitt and Scheiner 2004; Chown et al. 2007) and can be of particular importance when considering species interactions (Berg and Ellers 2010). Despite the highly dynamic character of the thermal regime at our field site, which is generally thought to favour phenotypic plasticity of the related traits (Agrawal 2001), we found no effects of the heating treatment on CT_max_ in any of the studied taxonomic groups. Such a result is inconsistent with a scenario of phenotypic plasticity of CT_max_. Previous studies have reported that plastic responses in traits related to thermal maxima are often less pronounced than those of thermal minima (e.g., Chown 2001; Jumbam et al. 2008; Alford et al. 2012; van Heerwaarden et al. 2016; see also the recent review on plasticity in extreme environments by Chevin and Hoffmann 2017), indicating that this limited plasticity of thermal maxima might be a general phenomenon among arthropods.

Animals can also behaviourally respond to changes in temperature to avoid adverse conditions (e.g., Kearney et al. 2009; Buckley et al. 2015; Duffy et al. 2015; Woods et al. 2015; MacLean et al. 2016). Horizontal or vertical movement in the soil allows animals to utilize small-scale spatial variability in micro-habitat thermal conditions. Even small absolute differences in temperatures between micro-habitat patches can be ecologically significant by reducing or increasing temperature by the few degrees that may determine survival or death (Scheffers et al. 2014; Kaspari et al. 2015; Pincebourde and Casas 2015). For instance, dense tussocks can reduce the amplitude of temperature fluctuations by several degrees, as we observed in our field site (Fig. S8), and hiding in this buffered microhabitat might prevent exposure of individuals to lethal high temperatures (Huey et al. 2012). To prevent variation caused by differences in microhabitats, we choose our plots to have homogeneous vegetation without such dense tussocks. A similar reduction in exposure to high temperatures may be achieved by vertical movement deeper into the soil (e.g., van Dooremalen et al. 2013). Although temperature differences down the soil profile can be large even over a few centimetres (Krab et al. 2015), we do not expect that our study organisms can fully utilize this temperature gradient. The vegetated beach ecosystem we studied has only limited possibilities for vertical stratification as the sandy soil is rather compact and often water locked. So both phenotypic and behavioural plasticity likely play a limited role in the patterns observed in our study.

In this study, we directly compared temperature sensitivity across trophic levels in a local terrestrial community. We observed interspecific differences in heat tolerance within a soil arthropod community, especially between the more temperature sensitive prey *I. riparia* (Collembola) and their more tolerant predators. As a consequence, during a heat wave the population size of prey might be reduced and negatively affect the growth rate or abundance of the predators, causing bottom–up effects in this food web. A severe reduction in Collembola availability could potentially also lead to a switch to a different prey species, including the possibility of intra-guild predation or cannibalism (Samu et al. 1999), and an associated change in community structure (Barton and Schmitz 2009; Rosenblatt and Schmitz 2016). In this study we focused specifically on a trait related to the effects of extreme temperatures, the Critical Thermal maximum (CT_max_). We argue that applying a trait-based approach allows for the formulation of predictions on how trophic interactions in food webs will be affected by extreme climatic events (sensu McGill et al. 2006; Violle et al. 2007). Including ecophysiological trait measurements, such as heat tolerance, in field studies on effects of climate extremes on community composition can therefore be a powerful method to point out direct effects of abiotic change at the species level, but can also help to reveal physiological mismatches between interacting species, which might shape the community dynamics on longer time scales.

## Electronic supplementary material

Below is the link to the electronic supplementary material.

Supplementary material 1 to 8 (DOCX 764 kb)
