# Supplementary material for: Heated communities: large inter- and intraspecific variation in heat tolerance across trophic levels of a soil arthropod community
Source: Oecologia. 2017 Dec 9;186(2):311–22. doi: 10.1007/s00442-017-4032-z (PMC5799326; doi:10.1007/s00442-017-4032-z)
Supplement: Supplementary file 1 — Supplementary material 1 to 8 (DOCX 764 kb) [file 442_2017_4032_MOESM1_ESM.docx]

**Electronic Supplemental Material**

**Heated communities: Large inter- and intraspecific variation in heat tolerance across trophic levels for a soil arthropod community**

Oscar Franken*^a^, Milou Huizinga^a^, Jacintha Ellers^a^, Matty P. Berg^a,b^

^a^ Section of Animal Ecology, Department of Ecological Science, Vrije Universiteit, Amsterdam, De Boelelaan 1085, 1081 HV Amsterdam, The Netherlands.

^b^ Groningen Institute for Evolutionary Life Sciences, Community and Conservation Ecology Group, University of Groningen, PO Box 11103, 9700 CC Groningen, The Netherlands.

*oscarfranken@gmail.com/ o.franken@vu.nl

Supplemental information 1: Location of field site with artificial heat wave treatments.

Supplemental information 2: Statistical justification of combining the two experimental periods.

Supplemental information 3: Experimental set-up to measure CT_max_ of soil arthropods.

Supplemental information 4: Equation to calculate CT_max_ values from the observed water temperature.

Supplemental information 5: Details of treatment effects on abiotic conditions.

Supplemental information 6: Factors excluded from main analysis

Supplemental information 7: Interspecific relation between CT_max_ and Body size for all taxonomic groups tested in our study.

Supplemental information 8: Buffering capacity of vegetation types

Supplemental information 1: Location of field site with artificial heat wave treatments.


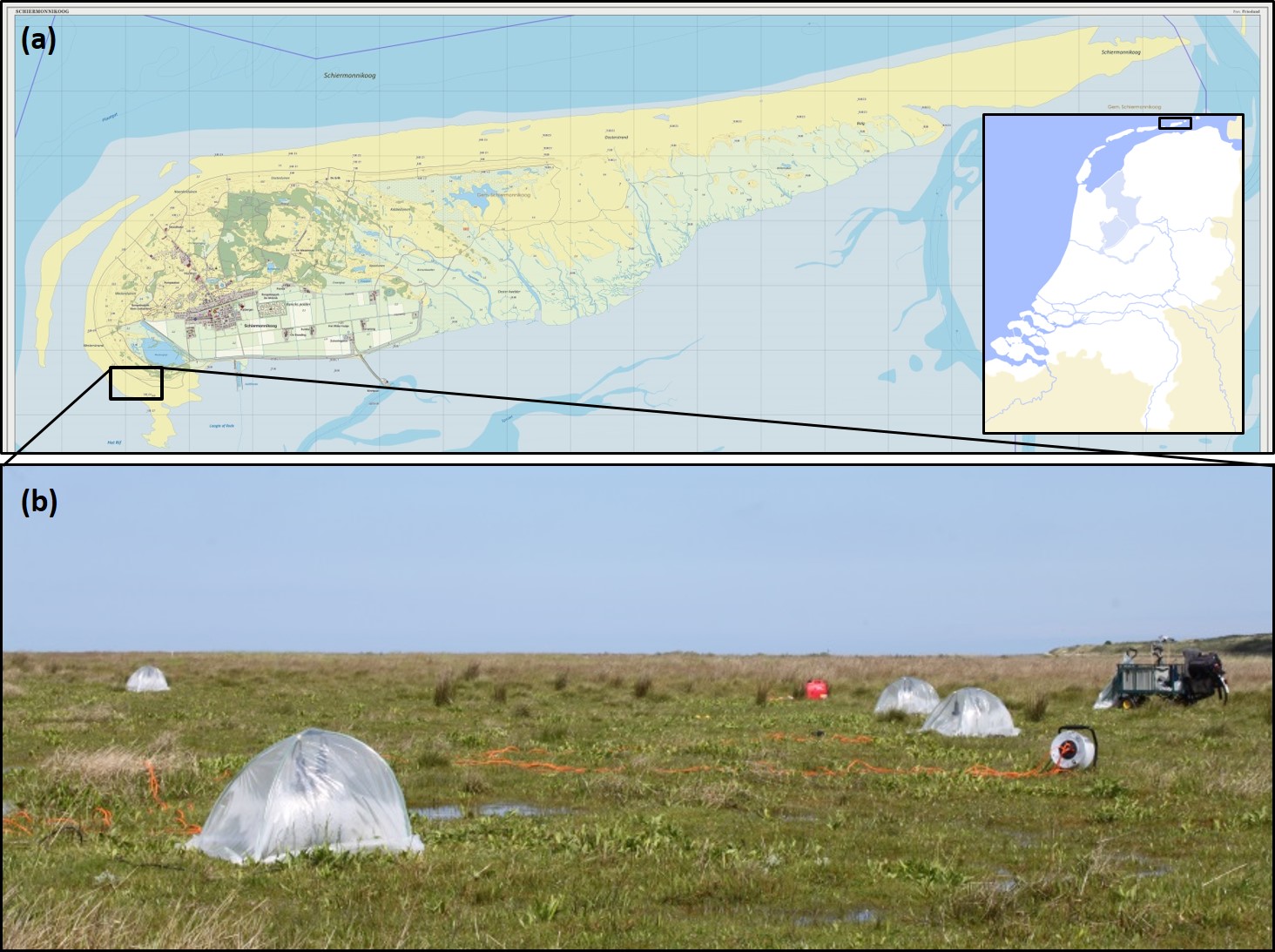


Fig. S1. Location (a) of our field site at the west side of the barrier Island of Schiermonnikoog, The Netherlands, and (b) experimental additional heat treatments in the field. Sources of images in panel (a): http://upload.wikimedia.org/wikipedia/commons/c/ce/Natuur-Schiermonnikoog-2014Q1.jpg and http://www.lesidee.nl/lif05/images/kaartNL2_1600.gif

Supplemental information 2: Statistical justification of combining the two experimental periods.

In the main text we describe that our preliminary analysis on the data from the first experimental period showed no effect of this single heat wave on the thermal sensitivity of the soil organisms. Therefore, in the second experimental period we applied two consecutive artificial heat waves of five days each to further increase thermal stress. By doing this, we are not able to disentangle the effects of the experimental period and the number of heat waves, therefore the combined effect of experimental period and number of heat waves was included as a random effect in the statistical analyses to account for any introduced variation by these combined effects.

In the statistical tests below, we show that only the factor Taxon significantly affected the fit of our model, also when analyzing the two experimental periods separately.

Experimental period 1:

*Analysis of Deviance Table (Type II Wald chisquare tests)*

*Response: CTmax*

*Chisq Df Pr(>Chisq)*

*Treatment 2.5338 1 0.1114*

*Taxon 615.1348 5 <2e-16 ****

*Treatment:Taxon 4.4138 5 0.4915*

Experimental period 2:

*Analysis of Deviance Table (Type II Wald chisquare tests)*

*Response: CTmax*

*Chisq Df Pr(>Chisq)*

*Treatment 0.2158 1 0.6423*

*Taxon 408.4067 5 <2e-16 ****

*Treatment:Taxon 6.8522 4 0.1439*

Important to note is that by combining these two experimental periods, we could compare more taxa within the same analysis, as some taxa were only present in sufficient numbers in either one of the Periods (i.e. *Erigone longipalpis* in period 1 and Heteroceridae in period 2). For the taxa that were present in both the Experimental periods, we did an additional analysis for each separate taxon to test the effect of the main factors Treatment and Experiment. The results of these analyses are given below per Taxon.

*Isotoma riparia:*

*Analysis of Deviance Table (Type II Wald chisquare tests)*

*Response: CTmax*

*Chisq Df Pr(>Chisq)*

*Treatment 2.1431 1 0.1432*

*Experiment 40.0905 1 2.425e-10 ****

*Treatment:Experiment 0.3302 1 0.5655*

*---*

*Signif. codes: 0 ‘***’ 0.001 ‘**’ 0.01 ‘*’ 0.05 ‘.’ 0.1 ‘ ’ 1*

*Oedothorax retusus:*

*Analysis of Deviance Table (Type II Wald chisquare tests)*

*Response: CTmax*

*Chisq Df Pr(>Chisq)*

*Treatment 0.3599 1 0.5486*

*Experiment 0.0424 1 0.8368*

*Treatment:Experiment 5.3432 1 0.0208 **

*---*

*Signif. codes: 0 ‘***’ 0.001 ‘**’ 0.01 ‘*’ 0.05 ‘.’ 0.1 ‘ ’ 1*

Juvenile Linyphiidae:

*Analysis of Deviance Table (Type II Wald chisquare tests)*

*Response: CTmax*

*Chisq Df Pr(>Chisq)*

*Treatment 0.7356 1 0.3911*

*Experiment 0.1542 1 0.6946*

*Treatment:Experiment 1.7582 1 0.1849*

Juvenile Lycosidae:

*Analysis of Deviance Table (Type II Wald chisquare tests)*

*Response: CTmax*

*Chisq Df Pr(>Chisq)*

*Treatment 0.2240 1 0.6360*

*Experiment 0.4502 1 0.5023*

*Treatment:Experiment 0.6703 1 0.4129*

Saldidae (No interaction tested, as there was insufficient replication in the second experimental period):

*Analysis of Deviance Table (Type II Wald chisquare tests)*

*Response: CTmax*

*Chisq Df Pr(>Chisq)*

*Treatment 0.0598 1 0.8068*

*Experiment 0.6079 1 0.4356*

For *I. riparia*, there was a significant effect of the Experimental period on CTmax and for *O. retusus* there was a significant interaction between Treatment and Experimental period. These are further investigated by contrasts’ analysis below:

*Isotoma riparia:*

*$lsmeans*

*Experiment lsmean SE df lower.CL upper.CL*

*1_time_wave 42.788 0.3649954 4.36 41.80666 43.76918*

*2_times_wave 44.097 0.3756443 4.81 43.11955 45.07419*

*Results are averaged over the levels of: Treatment*

*Confidence level used: 0.95*

*$contrasts*

*contrast estimate SE df t.ratio p.value*

*1_time_wave - 2_times_wave -1.308951 0.2087336 249.15 -6.271 <.0001*

*Oedothorax retusus:*

*$lsmeans*

*Treatment Experiment lsmean SE df lower.CL upper.CL*

*Control 1_time_wave 43.85476 0.9569524 37.42 41.91652 45.79299*

*Treatment 1_time_wave 46.37733 0.6928915 29.95 44.96216 47.79249*

*Control 2_times_wave 45.75236 0.5375434 5.46 44.40480 47.09992*

*Treatment 2_times_wave 45.49352 0.3359520 3.16 44.45445 46.53259*

*Confidence level used: 0.95*

*$contrasts*

*contrast estimate SE df t.ratio p.value*

*Control,1_ti - Treatment,1_ti -2.5225720 1.1050443 50.74 -2.283 0.1156*

*Control,1_ti - Control,2_ti -1.8976002 1.0180317 52.50 -1.864 0.2559*

*Control,1_ti - Treatment,2_ti -1.6387644 0.9876024 52.79 -1.659 0.3552*

*Treatment,1_ti- Control,2_ti 0.6249717 0.8335773 50.48 0.750 0.8763*

*Treatment,1_ti- Treatment,2_ti 0.8838076 0.7512626 52.31 1.176 0.6442*

*Control,2_tim - Treatment,2_ti 0.2588359 0.5733063 52.70 0.451 0.9691*

*P value adjustment: tukey method for comparing a family of 4 estimates*

For *I. riparia,* the differences in CTmax are significantly different between the two experimental periods, where in the first experimental period the CTmax is slightly lower (42.8 ± 0.36) than during the second experimental period (44.10 ± 0.38).

For *O. retusus* the outcome of the contrasts analysis was unexpected, as no significant results were found in any of the contrast-pairs.

Of the taxa that were present in both experimental periods, only *I. riparia* gave a significant result for the factor Experiment, where in the first experimental period the CT_max_ is slightly lower (42.8 ± 0.36) than during the second experimental period (44.10 ± 0.38). This small difference, together with the benefit of comparing more taxa within the same analysis, while still incorporating this variation as a random effect, leads us to believe that the main analysis presented in the manuscript is sufficiently warranted.

Supplemental information 3: Experimental set-up to measure CT_max_ of soil arthropods.


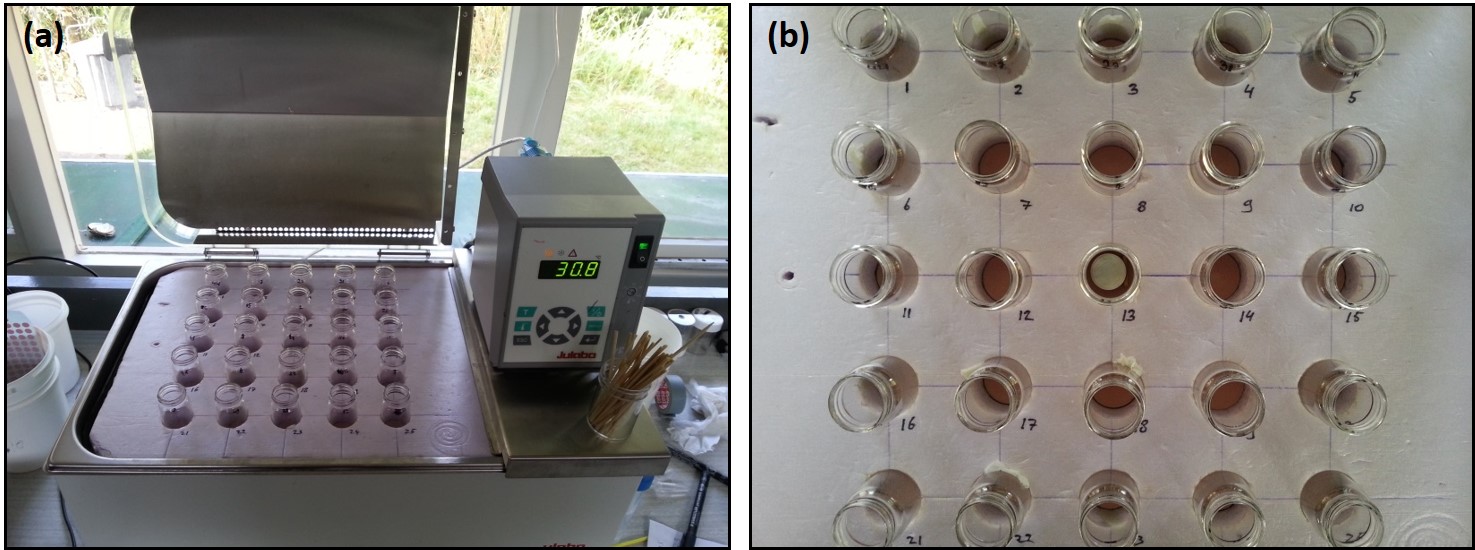


Fig. S3. Experimental set-up to measure the CT_max_ of soil fauna species. Panel (a) shows the water bath which gradually heats up with a steady increase of 0.33 ºC min^-1^ in the glass vials as monitored by an iButton temperature and humidity logger shown in the centre vial in panel (b). The remaining 24 vials each contained one individual during the trials.

Supplemental information 4: Equation to calculate CT_max_ values from the observed water temperature.

Fig. S4. Relationship between temperature of the water in the water bath and temperature in the vials placed in the water bath in which the animals were exposed. A temperature increase of 0.35°C min^-1^ of the water resulted in an temperature increase of 0.33°C min^-1^ in the vials. Data collected during previous experiments (not published). The slope of the curve was used to calculate the real temperature in the vials from the observable temperature of the water, see equation 1.

*CT_max_ = T_vial_ = 0.9074*T_water_ + 1.7151*

Equation 1. To calculate CT_max_ values from the recorded water temperature.

Supplemental information 5: Details of treatment effects on abiotic conditions.

Fig. S5. Heat treatment effects plotted per pair of subplots in (a), the first artificial heat wave, (b) the first and (c) the second week of two consecutive artificial heat waves. For each exposure week, both the average and maximum temperatures were significantly higher in the heat wave treatment. See table 2 for details.

Supplemental information 6: Factors excluded from main analysis


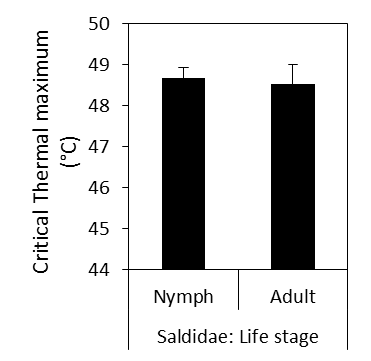

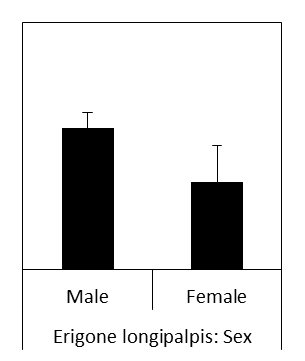

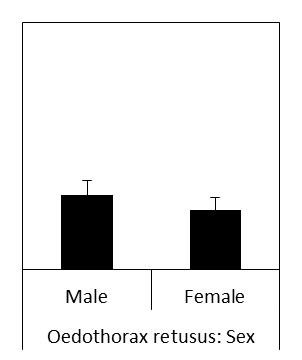


**(a)**

**(b)**

**(c)**

Fig. S6. Some factors in our analysis could only be recorded in a subset of the taxa investigated. In order to facilitate the analysis and subsequent interpretation of the results, we first tested for these specific taxa whether these factors significantly affected the fit of our statistical model. This was not the case for (a) the factor Life stage in Saldidae (*χ*^2^_(1)_ = 0.035, *P* = 0.85, Nymphs: *N* = 27, Adults: *N* = 4), or the factor Sex in both (b) *Erigone longipalpis* (*χ*^2^_(1)_ = 2.7314, *P* = 0.098, Male: *N* = 25, Female: *N* = 11) and (c) *Oedothorax retusus* (*χ*^2^_(1)_ = 0.43, *P* = 0.51, Male: *N* = 16, Female: *N* =41). Based on these results, these factors were not used in the main analysis.

Supplemental information 7: Interspecific relation between CT_max_ and Body size for all taxonomic groups tested in our study.


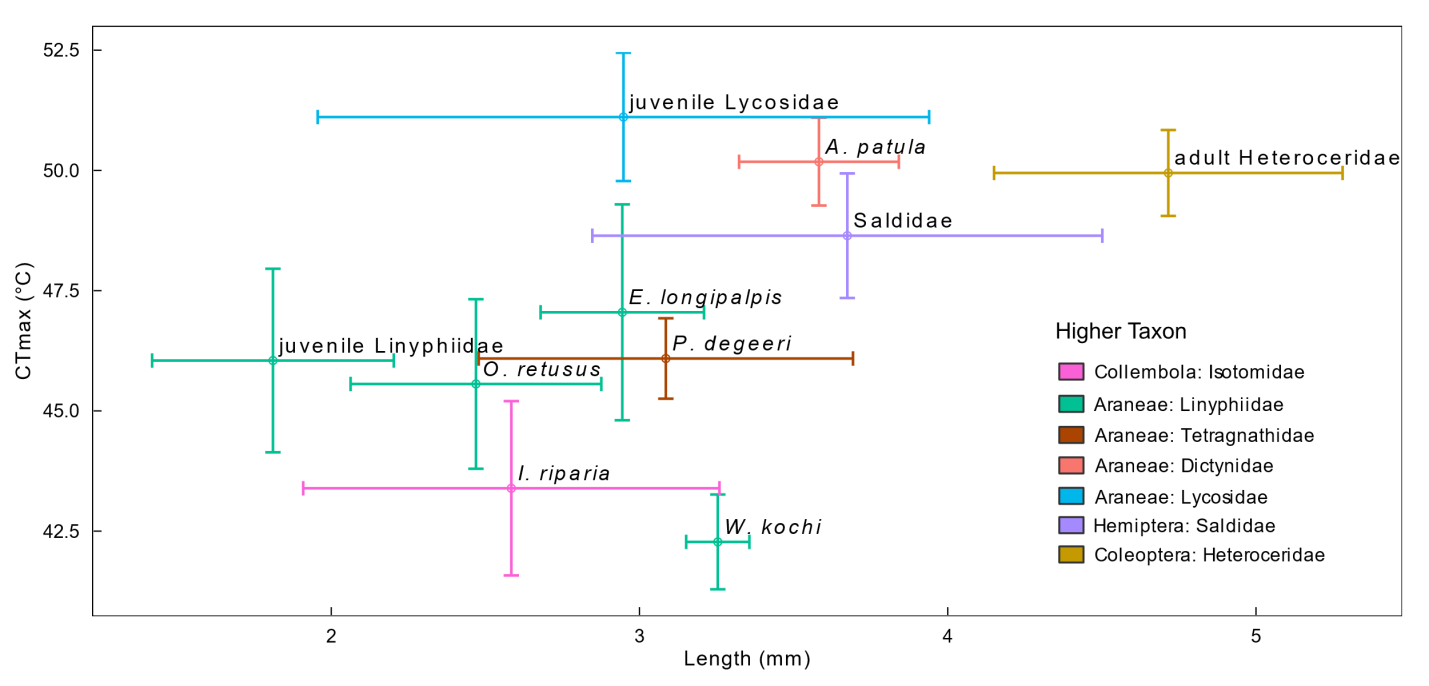


Fig. S7. Mean body size (mm ± St. deviation) and CT_max_ (ºC ± St. deviation) plotted for every taxonomic group in the analysis. A linear model was fitted through the data, weighted by the number of individuals within these taxa. The positive relation between the taxa was not significant. For full species names see Table 1.

Supplemental information 8: Buffering capacity of vegetation types


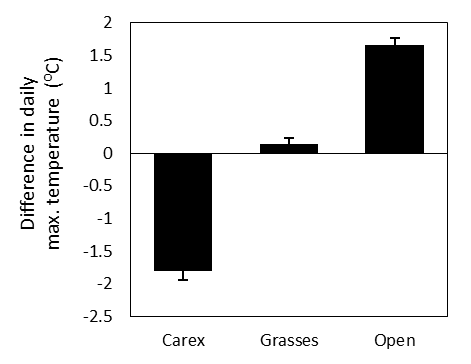


**(a)**


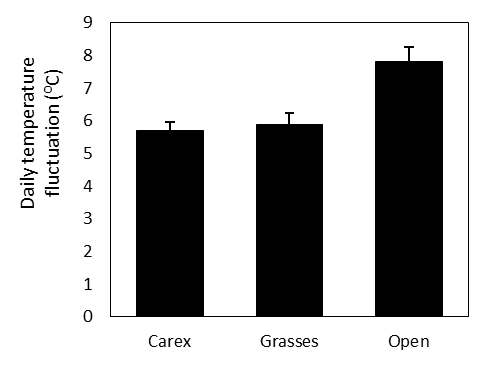


**(b)**

Fig. S8. Temperature buffering capacity of different vegetation types, with increasing complexity in tussock structure; “Carex” (a tall sedge vegetation consisting of dense tussocks), “Grasses” (a dense, medium tall grassy vegetation) and “Open” (bare soil with a microbial mat and sparse and short *Glaux maritima* plants. Both the difference in daily maximum temperature (a) and the absolute daily temperature fluctuation (b) are influenced by the vegetation types, and might provide some spatial heterogeneity in temperatures. Data was collected from 31^st^ July to 4^th^ November 2015.
